# Review on the phytochemistry and toxicological profiles of *Aloe vera* and *Aloe ferox*

**DOI:** 10.1186/s43094-021-00296-2

**Published:** 2021-07-21

**Authors:** Florence Nalimu, Joseph Oloro, Ivan Kahwa, Patrick Engeu Ogwang

**Affiliations:** 1 Pharm-Bio Technology and Traditional Medicine Centre of Excellence, Mbarara University of Science and Technology, Mbarara, Uganda; 2 Department of Pharmaceutical Sciences, Faculty of Medicine, Mbarara University of Science and Technology, P.O. Box 1410, Mbarara, Uganda; 3 Department of Pharmacology and Therapeutics, Faculty of Medicine, Mbarara University of Science and Technology, Mbarara, Uganda; 4 Department of Pharmacy, Faculty of Medicine, Mbarara University of Science and Technology, Mbarara, Uganda

**Keywords:** *Aloe vera*, *Aloe ferox*, *Aloe*, Phytochemistry, Toxicity, Review, Safety

## Abstract

**Background:**

*Aloe vera* and *Aloe ferox* have over the years been among the most sought-after *Aloe* species in the treatment of ailments worldwide. This review provides categorized literature on the phytochemical and scientifically proven toxicological profiles of *A. vera* and *A. ferox* to facilitate their exploitation in therapy.

**Main body of the abstract:**

Original full-text research articles were searched in PubMed, ScienceDirect, Research gate, Google Scholar, and Wiley Online Library using specific phrases. Phenolic acids, flavonoids, tannins, and anthraquinones were the main phytochemical classes present in all the two *Aloe* species. Most of the phytochemical investigations and toxicity studies have been done on the leaves. *Aloe vera* and *Aloe ferox* contain unique phytoconstituents including anthraquinones, flavonoids, tannins, sterols, alkaloids, and volatile oils. *Aloe vera* hydroalcoholic leaf extract showed a toxic effect on Kabir chicks at the highest doses. The methanolic, aqueous, and supercritical carbon dioxide extracts of *A. vera* leaf gel were associated with no toxic effects. The aqueous leaf extract of *A. ferox* is well tolerated for short-term management of ailments but long-term administration may be associated with organ toxicity. Long-term administration of the preparations from *A. vera* leaves and roots was associated with toxic effects.

**Short conclusion:**

This review provides beneficial information about the phytochemistry and toxicity of *A. vera* and *A. ferox* and their potential in the treatment of COVID-19 which up to date has no definite cure. Clinical trials need to be carried out to clearly understand the toxic effects of these species.

## Background

*Aloe* species (family Asphodelaceae) are among the most widely used plants over centuries for treating various ailments, for esthetic, and skincare [1]. The *Aloe* genus comprises over 430 species including *A. vera* and *A. ferox* among others [2]. These species have been reported to have pharmacological activities including anti-inflammatory, immunomodulatory, antibacterial, antifungal, antiviral, antiproliferative, antidiabetic, laxative, wound healing, moisturizing, anti-aging, and skin protection [3–5].

*Aloe* species are increasingly being incorporated into different cosmetic products, health drinks, foods, and beverages due to the abovementioned beneficial biological activities of the phytochemicals found mainly in the leaves.

These phytochemicals include polysaccharides, flavonoids, carbohydrates, coumarins, tannins, chromones, alkaloids, anthraquinones, organic compounds, pyrones, phytosterols, anthrones, sterols, vitamins, proteins, and mineral constituents [2, 5, 6]. The variation in concentration of these chemical constituents is based on the plant part used, extraction process, solvent, stage of growth, and plant source.

Though beneficial, some of these phytochemicals may be associated with toxic effects [7]. Many researchers have established potential toxicities as well as risks associated with some plants and vegetables particularly hepatotoxicity, nephrotoxicity, and cancer [8, 9]. Due to these risks, toxicological evaluation of medicinal plants has become one of the main concerns to assure their safe use [10, 11].

This review focuses on the phytochemistry and toxicology of *A. vera* and *A. ferox*, the two commercially popular species of *Aloe*. The present study will help in the standardization and quantification of the phytochemicals present in the *Aloe* species. It will also create awareness to the locals of the toxic effects that may be associated with the use of these species as medicine and future studies in humans.

## Main text

The search was made in the databases of PubMed, ScienceDirect, Research gate, Google Scholar, and Wiley Online Library using the phrases “Genus *Aloe*,” “*A. vera*,” “toxicology of *Aloe* species,” “acute and subacute toxicity of *Aloe* species,” safety, “*A. ferox*,” and “phytochemistry of *Aloe* species.” Published original full-text articles in English language on phytochemistry and toxicity of the *Aloe* species were retrieved.

### Phytochemistry of the *Aloe* species

*Aloe vera* and *Aloe ferox* contain vast phytochemical classes including anthraquinones, chromones, anthrones, phenolic compounds, flavonoids, tannins, steroids, and alkaloids which contribute to their different pharmacological activities. The structures of the individual compounds are included (Figs. 1, 2, 3, 4, 5, 6, 7, 8, 9, 10, 11, 12, 13, 14, 15, 16, 17, 18, 19, and 20). More information on phytochemistry is summarized in Tables 1, 2, and 3.

**Fig. 1:**
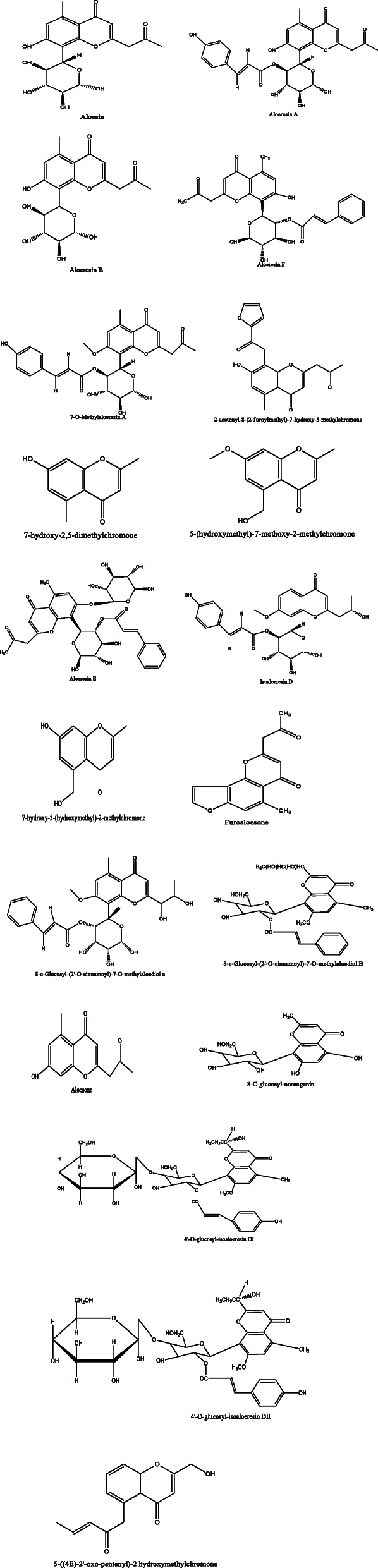
Chemical structures of chromones isolated from *A. vera* and *A. ferox*

**Fig. 2:**
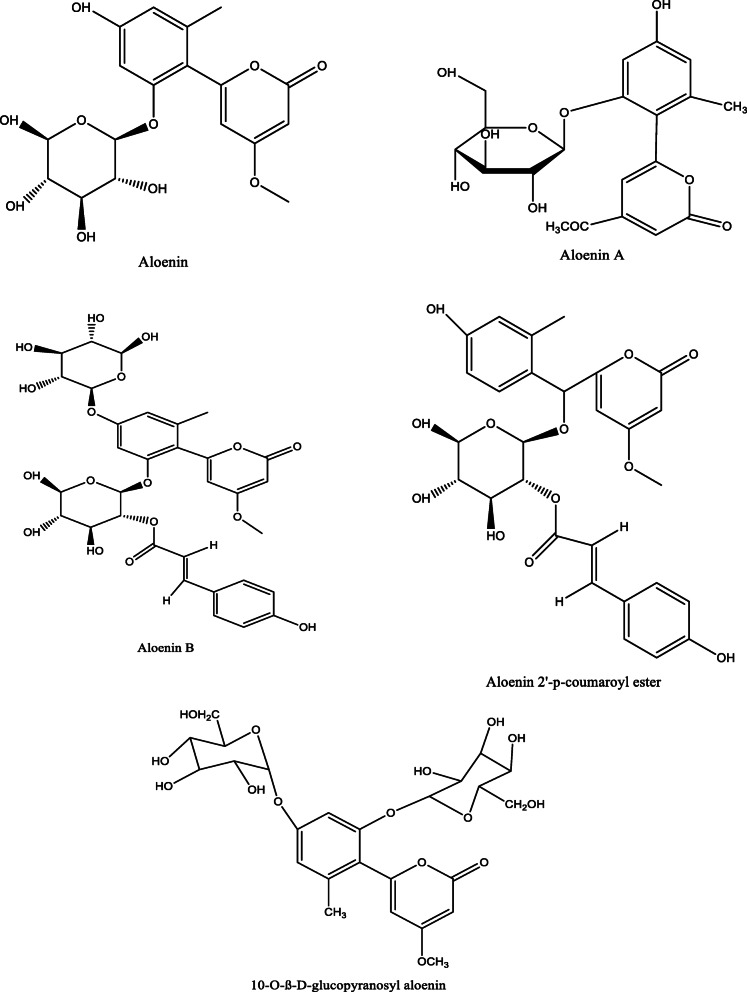
Chemical structures of phenyl pyrones isolated from *A. vera* and *A. ferox*

**Fig. 3:**
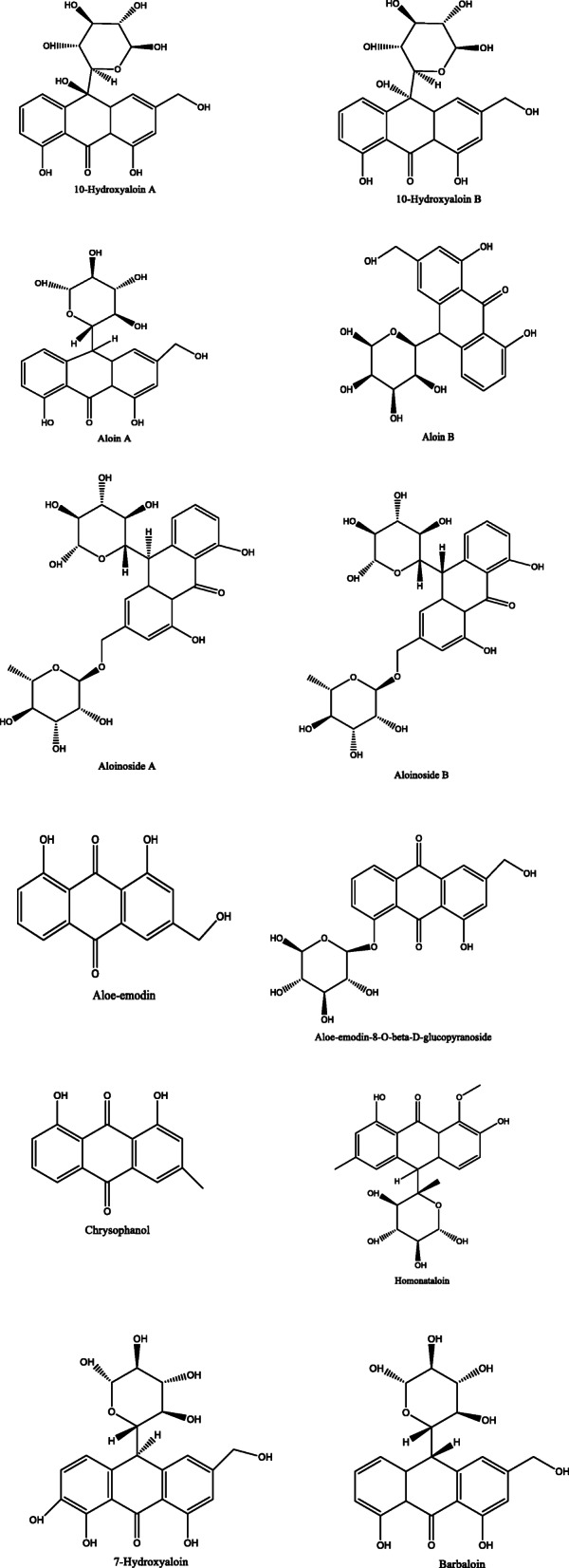
Chemical structures of anthrones isolated from *A. vera* and *A. ferox*

**Fig. 4:**
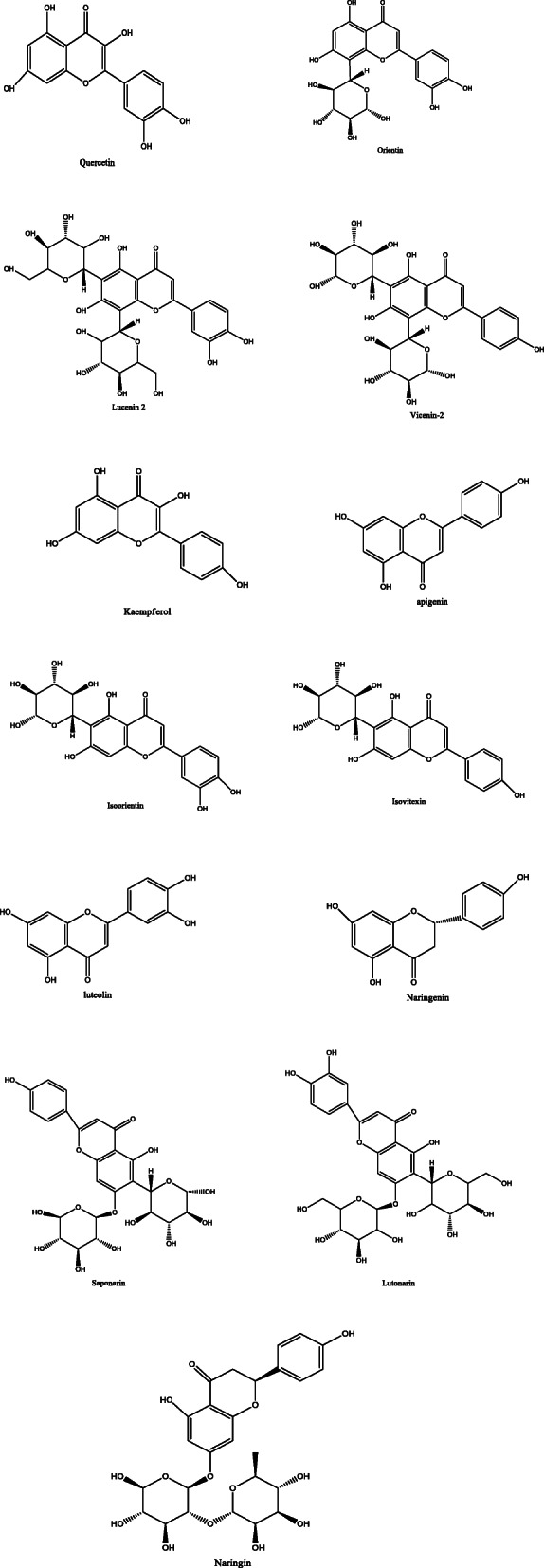
Chemical structures of flavonoids isolated from *A. vera* and *A. ferox*

**Fig. 5:**
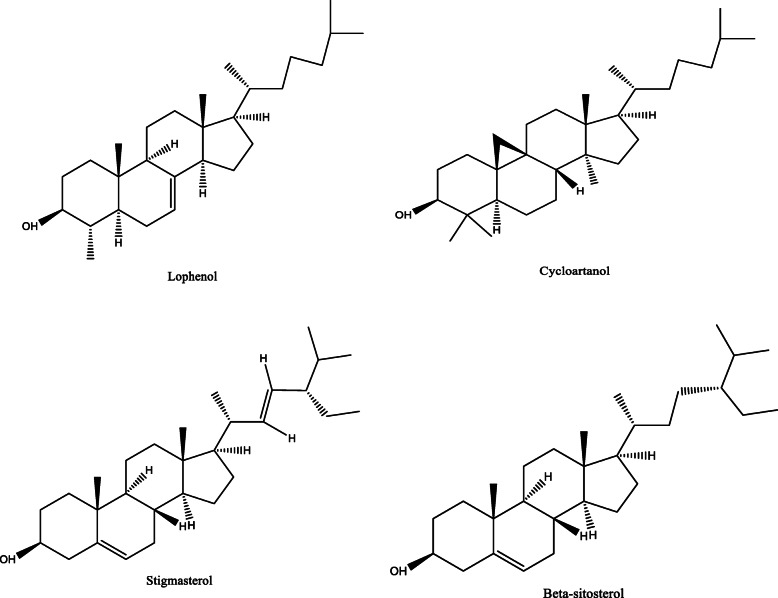
Chemical structures of sterols isolated from *A. vera* and *A. ferox*

**Fig. 6:**
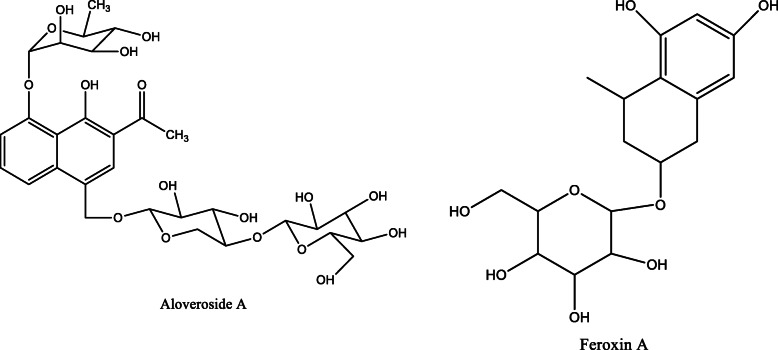
Chemical structures of the naphthalene derivatives isolated from *A. vera* and *A. ferox*

**Fig. 7:**
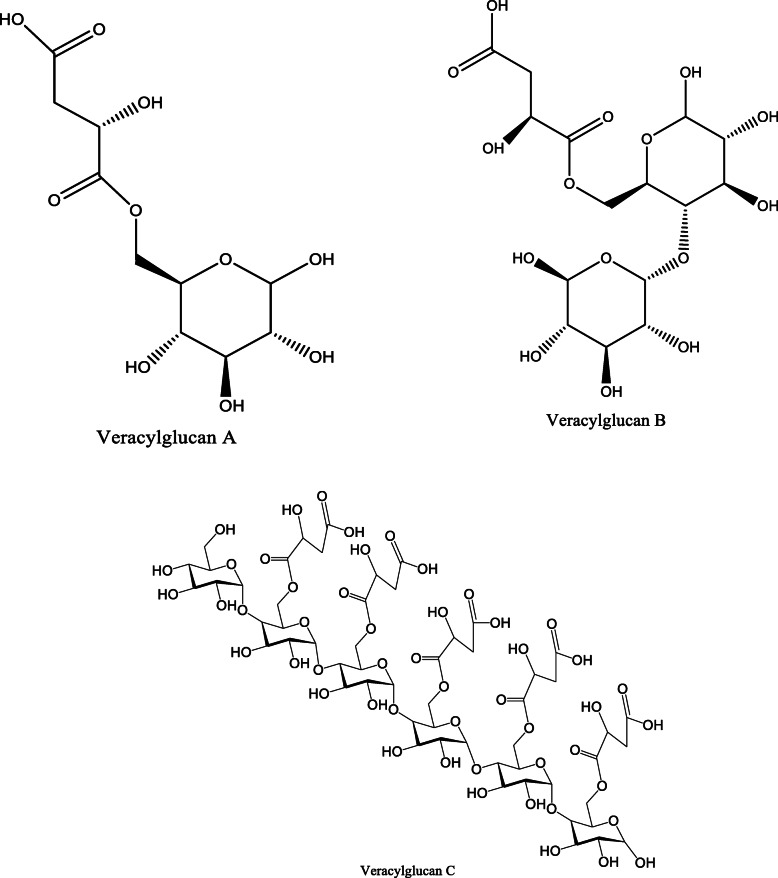
Chemical structures of the maloyl glucans isolated from *A. vera*

**Fig. 8:**
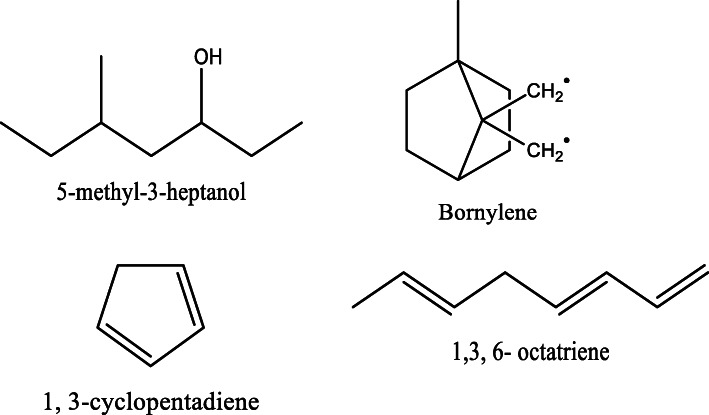
Chemical structures of volatile oils isolated from *A. ferox*

**Fig. 9:**
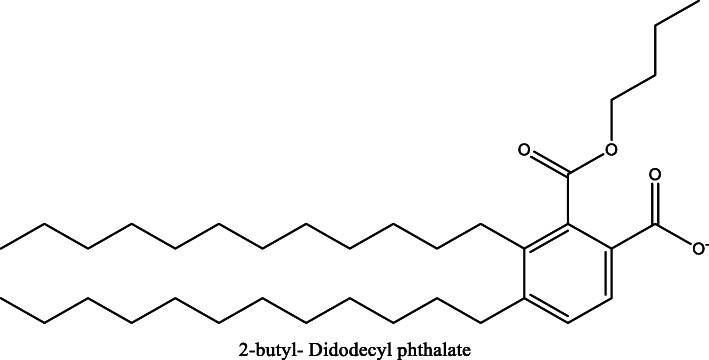
Chemical structure of an ester isolated from *A. vera*

**Fig. 10:**
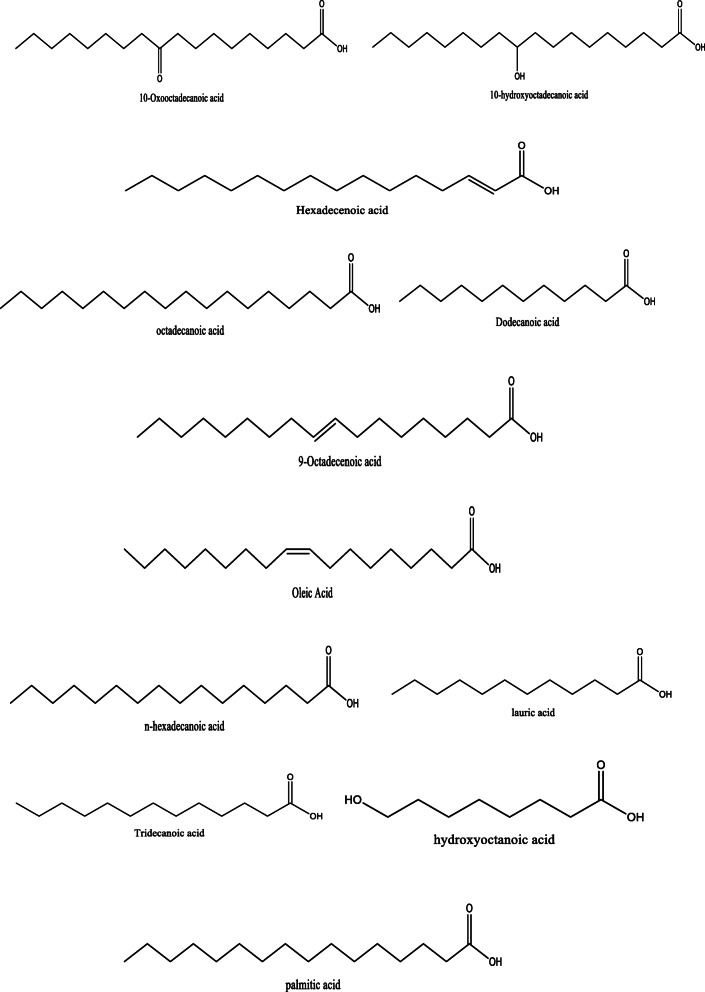
Chemical structures of fatty acids isolated from *A. vera* and *A. ferox*

**Fig. 11:**
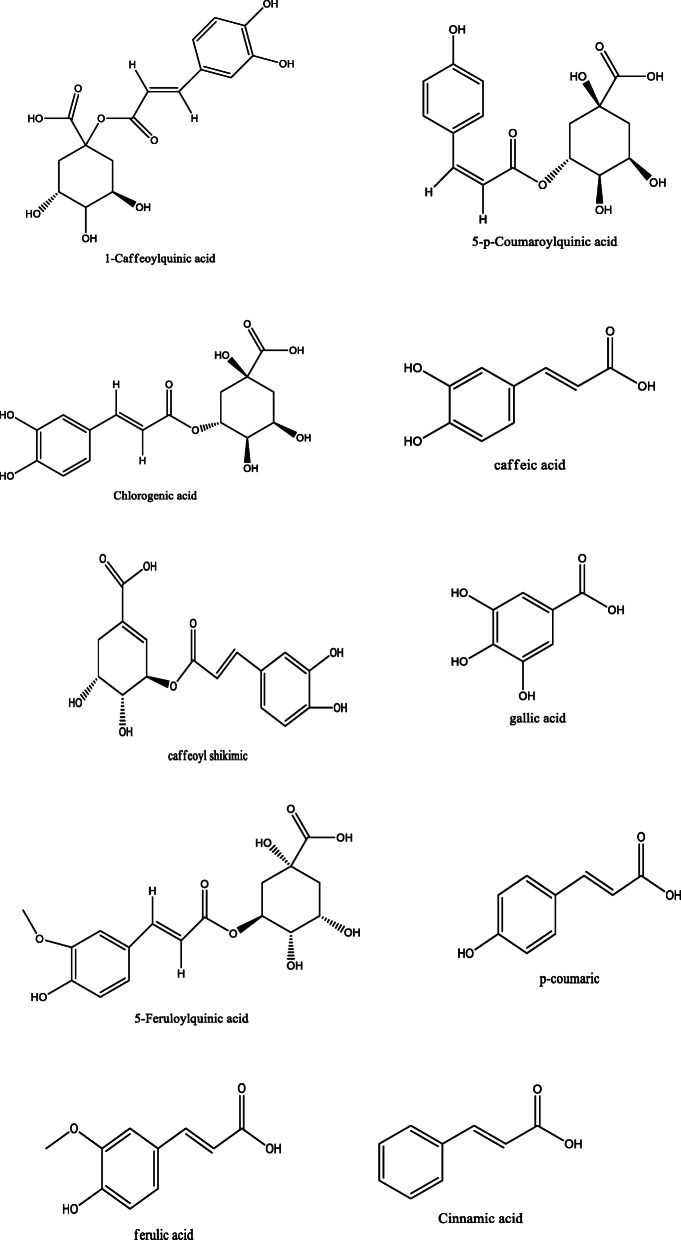
Chemical structures of phenolic acids isolated from *A. vera* and *A. ferox*

**Fig. 12:**
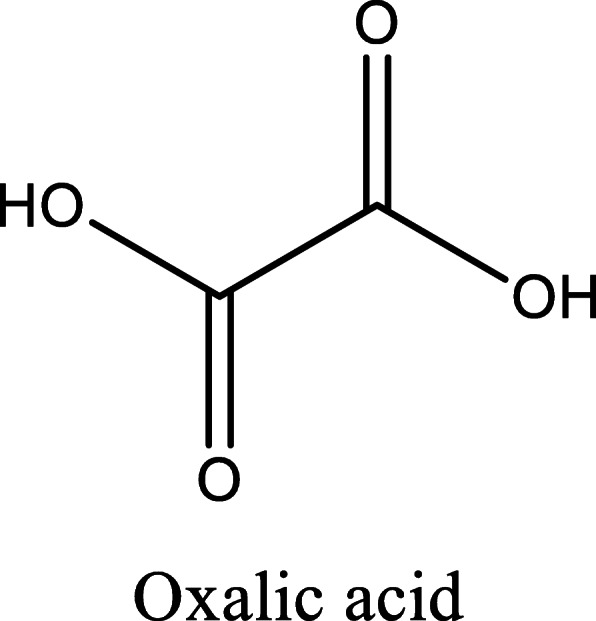
Chemical structure of a dicarboxylic acid isolated from *A. vera*

**Fig. 13:**
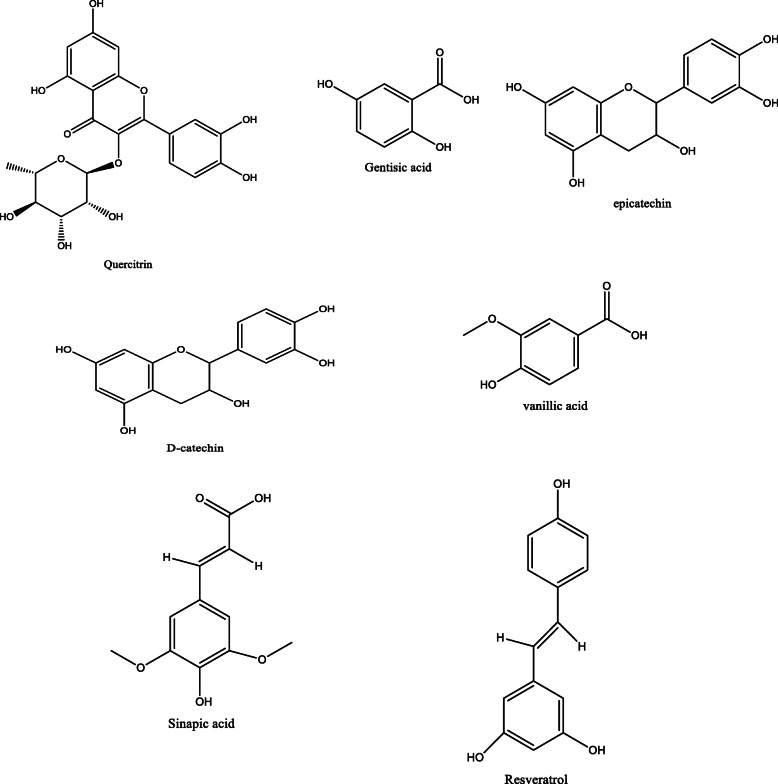
Chemical structures of phenolic compounds isolated from *A. vera* and *A. ferox*

**Fig. 14:**
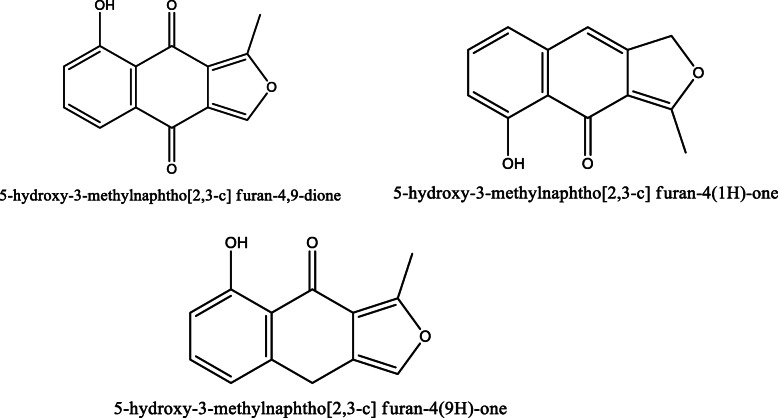
Chemical structures of naphtho [2, 3-c] furan-4, 9-dione derivatives isolated from *A. ferox*

**Fig. 15:**
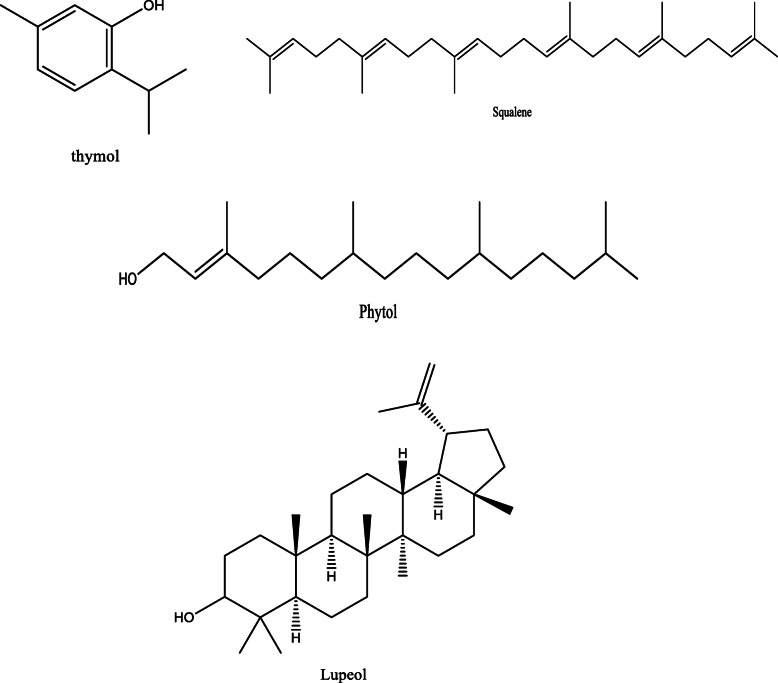
Chemical structures of some terpenoids isolated from *A. vera* and *A. ferox*

**Fig. 16:**
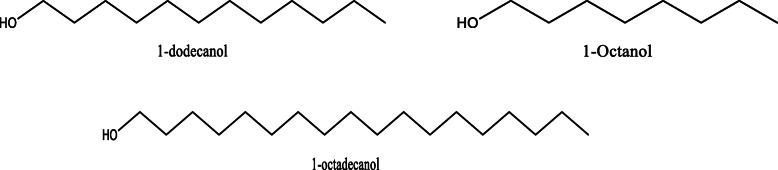
Chemical structures of some alcohols isolated from *A. vera* and *A. ferox*

**Fig. 17:**
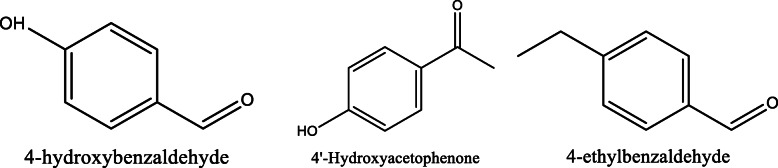
Chemical structures of some aldehydes isolated from *A. vera* and *A. ferox*

**Fig. 18:**
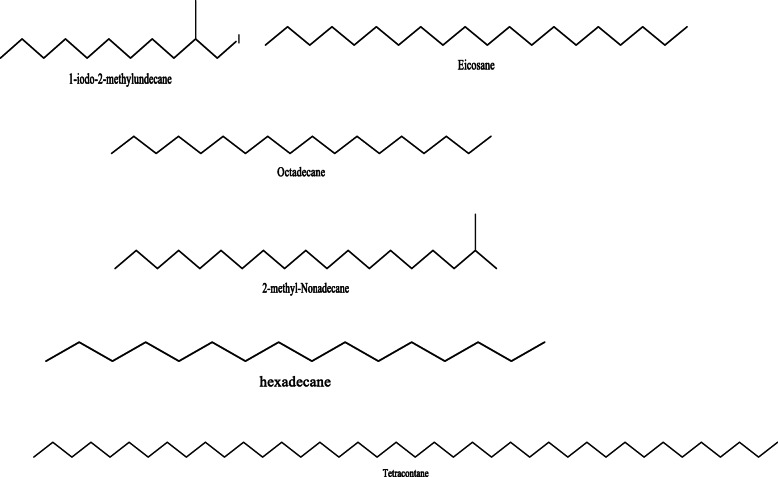
Chemical structures of some alkanes isolated from *A. vera* and *A. ferox*

**Fig. 19:**
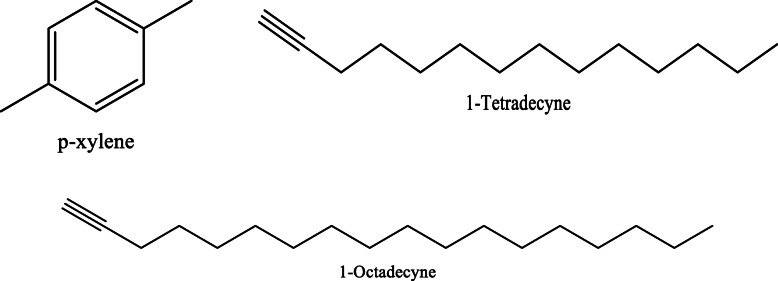
Chemical structures of some alkynes isolated from *A. vera* and *A. ferox*

**Fig. 20:**
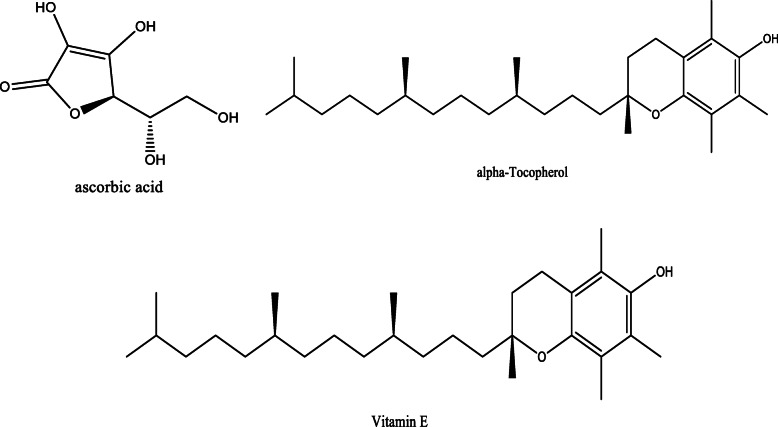
Chemical structures of some vitamins isolated from *A. vera* and *A. ferox*

**Table 1:** Phytochemical profile of whole leaves and flowers of *Aloe vera*

Plant part	Phytochemicals present	Method of extraction and solvent used	Method of detection	Ref
Fresh leaves	**Phenolic acids;** caffeoylquinic acid hexoside and 3,4-O-(E) caffeoyl feruloyl quinic acid **Anthraquinones;** Aloeresin E, isoaloeresin D, and 2'-O-feruloylaloesin **Flavonoids;** Orientin, vicenin II, and Lucenin II	Cold percolation (methanol)	HPLC–MS	[12]
	Phenols, Alkaloids, saponins, and sterols	Cold maceration (Hexane)	Phytochemical screening and TLC	[13]
	Saponins, sterols, and phenols	Cold maceration (Ethyl acetate)		
	Alkaloids, saponins, sterols, flavonoids, and phenols	Cold maceration (Methanol)		
	Alkaloids, tannins, sterols, flavonoids, and phenols	Cold maceration (Aqueous)		
	**Chromones**; aloesin, 8-C-glucosyl-7-O-methyl-(S)-aloesol and isoaloeresin D. **Phenyl pyrones**; aloenin and aloenin B **Anthrones**; Aloe emodin, aloin A and B, 8-O-methyl-7-hydroxyaloin A and B, and 10-hydroxyaloin A	Sonication (ethanol)	HPLC	[14]
	Sinapic acid, chlorogenic acid, aloin, aloe-emodin 8-O-beta-D-glucopyranoside, catechin, and epicatechin	Blended with 80% chilled acetone	HPLC	[15]
	Cardiac glycosides, steroids, flavonoids, reducing sugar, phenolic compounds, terpenoids, carbohydrates, amino acids, tannins, and saponin glycosides	Cold maceration (methanol and ethanol) Hot maceration (water)	Phytochemical screening	[16]
	Dietary fiber (mannan), malic acid, α-tocopherol, phenolic compounds, and apigenin glycoside derivatives	Soxhlet extraction (petroleum ether) Maceration (ethanol: water)	Uv-vis, MS	[17]
	Phytosterols (β-sitosterol)	N/A	GC-MS	[18]
	**Aldehydes**; 4-ethyllbenzaldehyde and benzene acetaldehyde **Acids**; lauric acid, palmitic acid **Carboxylic acids**; hydroxyoctanoic acid derivative, octadecanoic acid **Alkanes**; hexadecane derivative	Maceration (hexane)	GC-MS	[19]
	Terpenoids, Tannins, Flavonoids, resins, anthraquinones, saponins, glycosides, acidic compounds, lignin, semi anthraquinone like derivatives, polysaccharides, vitamin B complex, phenol-chromones, and chromones	Dissolution with 95% ethanol	Phytochemical screening and HPLC	[20]
	Alkaloids, anthraquinones, terpenes, phenols, tannins, coumarins, and flavonoids	Sonication (dichloromethane and methanol)	Phytochemical screening	[21]
Dried leaves	Alkaloids, phenols, flavonoids, saponins, glycosides, reducing sugars, phenolic compounds, tannins, steroids, and terpenoids	Cold percolation (methanol)	Phytochemical screening	[22]
	Flavonoids, tannins, and saponins **Terpenoids**; Squalene, phytol, and lupeol **Alkynes**; 1-Tetradecyne and 1-Octadecyne **Carboxylic acids**; Tridecanoic acid and n-Hexadecanoic acid **Alkanes**; 1-lodo-2-methylundecane, eicosane, octadecane, 2-methyl nonadecane, and tetracontane, 3,5,24-trimethyl-C **Fatty acids**; Oleic acid **Dicarboxylic acid**; Oxalic acid **Alcoho**l; 1-Octanol **Ester**; 2-butyl- didodecyl phthalate **Vitamins**; α-Tocopherol and vitamin E **Sterols**; β-Sitosterol	Soxhlet extraction (distilled water, ethanol, acetone solution)	Phytochemical screening and GC-MS	[23]
	Anthraquinones, tannins, flavonoids, saponins, squalene, oleic acid, dodecanoic acid, p-xylene, and n-hexadecanoic acid	Maceration (Water)	Phytochemical screening and GC-MS	[24]
	Saponins, phytosterols, terpenoids, alkaloids, flavonoids, carbohydrates, proteins, phenols, and carbohydrates	Soxhlet extraction (80% ethanol)	Phytochemical screening and GC-MS	[25]
	tannins, flavonoids, terpenoids, carbohydrates, and alkaloids	Soxhlet extraction (chloroform) Maceration (water)	Phytochemical screening	[26]
Dried Flowers	**Phenolic compounds;** Quercitrin, gentisic acid, and epicatechin	Maceration (methanol)	Reverse Phase-HPLC	[27]
	Coumarin, gallic acid, caffeic acid, D-catechin, vanillic acid, narigenin, resveratrol, cinnamic acid, thymol, quercetin, and naringin	Maceration (70% ethanol)	HPLC	[28]
	**Phenolic acids**; Chlorogenic, caffeic, 5-*p*-coumaroylquinic, caffeoyl shikimic, 5-feruloyl quinic, 5-*p*-cis-coumaroylquinic, *p*-coumaric, and ferulic acids **Flavonoids**; luteolin, apigenin, quercetin, isoorientin, isovitexin, kaempferol, saponarin, and lutonarin **Anthranoids**; Aloe emodin	Ultrasonication (methanol)	HPLC-DAD and HPLC-MS/MS	[29]

**Table 2:** Phytochemical profile of the gel, skin, powder, and extracts from *A. vera* leaves

Plant preparation used	Phytochemicals present	Method of extraction and solvent used	Method of detection	Ref
Crude herbal extract	Alkaloids, free anthraquinones, amino acids, saponins, tannins, triterpenoids, steroids, glycosides, and flavonoids	N/A	Phytochemical screening and TLC	[30]
Ethanol herbal extract	6-phenyl-2-pyrone derivatives (*p*-coumaroyl aloenin and aloenin A), naphthalene derivatives (aloveroside A), and anthraquinones.	N/A	TLC, HPLC, MS, IR, and NMR	[31]
Leaf exudate	Homonataloin, aloesin, aloenin, barbaloin, aloinosides A&B, and aloesone	Exudation into methanol	TLC	[32]
	**Chromones**; aloesin, 8-C-glucosyl-(R)-aloesol, 8-C-glucosyl-7-O-(S)-methylaloesol, and 5-((S)-2β-oxo-4’-hydroxypentyl-2(-glucopyranosyl-oxymethyl) chromone. **Phenyl pyrones;** 10-O-d-glucopyranosyl aloenin, aloenin, aloenin B, and aloenin-2’-*p*-coumaroyl ester **Anthrones**; 10-hydroxyaloin B, 10-hydroxyaloin A, aloin B, aloin A, aloinoside B, and aloinoside A **Anthraquinone**; Aloe emodin **Naphthalene derivative**; Aloveroside B	Ultrasonic extraction (methanol and water)	HPLC-DAD and LCMS-IT-TOF	[33]
	Free and glycosylated chromones: Aloesin and aloeresin A Anthraquinones: Aloin and aloe emodin	Sonication (methanol: acetone: ethyl acetate)	Colorimetric assays, TOF-MS	[34]
	Saponins, flavonoids, and tannins	Soxhlet (petroleum ether: chloroform: ethanol)	Phytochemical screening	[35]
Leaf gel	**Fatty acids**; hexadecanoic acid, octadecanoic acid, and 9-octadenoic acid **Sterols**; Sitosterol, and stigmasterol Alcohols; 1-octadecanol, 1-dodecanol **Alkanes**; debocane, tricosane, and 4-methyl, 1-(phenylthioxomethyl)piperidine	Maceration (ethanol)	GC-MS	[36]
	Chromones; 8-C-glucosyl-(2’-*O*-cinnamoyl)-7-*O*-methylaloediol A and B, 8*-C*-glucosyl-noreugenin, 4’-*O*-glucosyl-isoaloeresin DII, and 4’-*O*-glucosyl-isoaloeresin DI	(Ethanol)	HPLC and NMR	[37]
	Phytosterols; cycloartanol, lophenol, 24-ethyl-lophenol, 24-methyl-lophenol, and 24-methylene-cycloartanol	Trichloromethane and methanol	Column chromatography, NMR	[38]
	Maloyl glucans; Veracylglucan A, B, and C		NMR, ESIMS, MALDI-TOF-MS, and capillary electrophoresis.	[39]
	Pyrocatechol, ascorbic acid, coumaric acid, and *p*-coumaric acid	Cold maceration (ethanol and methanol)	Solvent fractionation, TLC, and GC-MS	[40]
	Alkaloids, aldehydes, phytosterols, pyrimidines, phenolic acids/polyphenols, fatty acids, alkanes, organic acids, alcohols, dicarboxylic acids, ketones, and indoles	Blended with 95% ethanol and centrifuged	GC-MS	[41]
	Carbohydrates, resins, reducing sugars, glucuronic acid, pentose derivatives, acetylated mannan, galactoglucoarabinomannan, glucomannone, and monosaccharides (alverose)	Extraction with ethanol	Phytochemical screening and HPLC	[20]
	Cardiotonic glycosides, anthraglycosides, mucilages, and reducing sugars	Extraction with water	Phytochemical screening	[42]
	Sterols type ∆^5^ and anthraquinones	Soxhlet (Chloroform)	Phytochemical screening	
	Triterpenoids, carbohydrates, saponins, anthraquinones, and naphthoquinones	Soxhlet extraction (Ethanol)	Phytochemical screening	
Leaf skin	Steroids, tannins, terpenoids, catechin, carotenoids, and anthraquinones	Maceration (ethanol)	Phytochemical screening	[43]
	**Phenolic compounds**; Sinapic acid, catechin, and quercetin	Maceration (methanol)	RP-HPLC	[28]
Leaf Powder	Chromones; Aloenin B, 5-(hydroxymethyl)-7-methoxy-2-methylchromone, aloin A & B, aloe emodin, 5-((4E)-2'-oxo-pentenyl)-2 hydroxymethylchromone, 7-hydroxy-5-(hydroxymethyl)-2-methylchromone, and 10-hydroxyaloin A &B	Ultrasonication (70% methanol)	UV, IR, 1D and 2D NMR, and High-Resolution Mass Spectrometry (HRMS)	[44]
Resin	Aloeveraside A and B, benzene derivatives, terpenoids, anthraquinones, coumarins, anthraquinone glycosides, quinones, polypodane-type, and ferroxidin	Cold maceration (methanol)	TLC, NMR, IR, and MS	[45, 46]

**Table 3:** Phytochemical profile of *Aloe ferox*

Plant part/preparation used	Phytochemicals present	Method of extraction and solvent used	Method of detection	Ref
Fresh leaves	**Phenolic acids;** caffeoylquinic acid hexoside and 3,4-O-(E) caffeoyl feruloyl quinic acid **Anthraquinones;** Aloeresin E, isoaloeresin D, and 2'-O-feruloylaloesin **Flavonoids;** Lucenin II, vicenin II, and orientin	Cold percolation (methanol)	HPLC-MS	[12]
	Sinapic acid, catechin, chlorogenic acid, aloe-emodin-8-O-beta-D-glucopyranoside, aloin, and epicatechin	Blended with 80% chilled acetone	HPLC	[15]
Dried leaves	Aloe emodin, aloin A, and chrysophanol	Maceration (water)	Vacuum liquid fractionation, column chromatography	[47]
	Phenols, saponins, alkaloids, flavonoids, proanthocyanidins, flavonols, and tannins	Cold maceration (distilled water, acetone, methanol, and ethanol)	Phytochemical screening	[48]
	Condensed tannins, flavonoids, and gallotannins	Extraction by sonication (methanol) followed by successive extraction with petroleum ether, dichloromethane, and ethanol)	Phytochemical screening	[49]
Dried leaf latex	Naphtha [2,3-c] furan derivatives; 5-hydroxy-3-methylnaphtho[2,3-c] furan-4,9-dione and 5-hydroxy-3-methylnaphtho[2,3-c] furan-4(1*H*)-one, anthraquinones, and 5-hydroxy-3-methylnaphtho[2,3-c] furan-4(9*H*)-one	Dissolution in water	X-ray analysis and spectroscopy	[50]
Leaf resin	hydroxyanthracene derivatives (aloin)	N/A	TLC	[51]
Leaf juice	Volatile oils; 5-methyl-3-heptanol, bornylene, 1, 3-cyclopentadiene, 3, 6 octatriene, and 3-cyclohexane-1-hetanol	Hydro distillation (water)	GC-MS	[52]
Dried exudate	**Free and glycosylated chromones;** Aloeresin B & F and 7-*O*-methyl aloeresin **Naphthalene derivative;** feroxin A **Anthraquinones;** hydroxyaloin and 8-*O*-Methyl- 7-hydroxyaloin	Sonication (methanol, acetone, and ethyl acetate mixture)	Colorimetric assays, Q-**TOF**-**MS**	[34]
	Aloe emodin, furoaloesone, *p*-hydroxybenzaldehyde, 10-oxooctadecanoic acid, *p*-hydroxyacetophenone, pyrocatechol, 7-hydroxy-2,5-dimethylchromone, 10-hydroxyoctadecanoic acid, 2-acetonyl-8-(2-furoylmethyl)-7-hydroxy-5-methylchromone, and methyl 10-hydroxyoctadecanoate,	Maceration (hexane and aqueous acetone)	Solvent partitioning, column chromatography, TLC, NMR, and MS	[53]
Roots	Phenols, alkaloids, flavonoids, tannins, flavonols, and saponins	Maceration (water)	Phytochemical screening	[54]
Leaf gel	Alkaloids, phenolic acids/polyphenols, phytosterols, organic acids, fatty acids, indoles, alkanes, alcohols, pyrimidines, aldehydes, dicarboxylic acids, and ketones	Blended with 95% ethanol and centrifuged	GC-MS	[55]

### Acute toxicity

According to Celestino et al. [51], *A. ferox* resin at a dose of 5000 mg/kg caused moderate diarrhea and reduced motor activity after 1 h post administration in Wistar rats.

Studies on both the methanolic and supercritical carbon dioxide extracts of *A. vera* leaf gel showed no treatment-related mortalities or changes in all the investigated parameters in rats [56, 57].

Aqueous leaf extracts of *A. vera* at doses of 200, 400, and 600 mg/kg and *A. ferox* at doses 500, 100, 200, and 400 mg/kg did not cause any toxic effects or mortality in all the treated animals [58–60]. Likewise, no toxic effects were observed when male Wistar rats were treated with an ethanolic extract of *A. vera* roots at doses of 100, 200, and 400 mg/kg [61].

Ethanolic, acetone, and aqueous extracts of *A. ferox* roots and leaves caused death of nauplii of the brine shrimps at concentrations above 0.5 mg/ml [62]. Similarly, a herbal extract of *A. vera* at concentrations of 0.01, 0.1, and 1 mg/ml was toxic to the nauplii of the brine shrimps [63]. A hydroalcoholic extract of *A. vera* leaves caused mortality at 2560–5120 mg/kg within 36–48 h in Kabir chicks [64]. A study by Shah et al. [65] revealed that an ethanolic extract of *A. vera* leaves caused reduced motor activity at doses of 1000 and 3000 mg/kg in male Swiss albino mice.

### Subacute toxicity

Administration of *Aloe vera* product (UP780), *A. vera* leaf juice, and gel for 14 days caused no harmful effects in rats and mice [58, 66, 67]. Wintola et al. [68] and Kwack et al. [69] reported similar results when *A. vera* leaf powder and *A. ferox* aqueous leaf extract were separately administered to rats.

A study by Koroye et al. [70] showed that administration of *Aloe vera* plus (GNLD) twice daily at volumes of 0.2, 0.4, and 0.8 cm^3^ for 14 and 28 days caused histological variations in the kidney tissues of the treated Wistar rats. A study by Sodani [71] displayed that the administration of 0.02 cm^3^ of *A. vera* leaf juice to male Swiss Webster mice over 21 days caused pathological effects on the kidneys.

In other studies, *Aloe vera* health drinks A and B administered over 28 days caused slight weight reduction and increase in white blood cell, red blood cell count, liver enzymes, serum urea, and creatinine levels in the rats given a volume of 1.0 cm^3^ [72]. *A. vera* leaf powder at a dose of 400, 1200, and 2000 mg/kg caused a significant reduction in white blood cell count and pigmentation of the kidneys in Sprague-Dawley rats [73].

Elevation in red blood cells, platelet count, hypertrophy of lungs, heart, and kidney and necrosis of spermatogenic cells was observed when an aqueous leaf extract of *A. ferox* at doses of 50, 100, 200, and 400 mg/kg was administered to Wistar rats for 14 days [59]. A decrease in the size of tubules, germ cell debris, and picnotic cells in the testes and testosterone was seen when *A. vera* gel product was administered for 28 days to male Swiss albino mice at the highest dose [74].

A study by Bala et al. [75] displayed that an aqueous gel extract of *A. vera* caused histopathological alterations in male Balb/c mice at 100 and 250 mg/kg.

### Sub-chronic and chronic toxicity

A study by Saritha and Anilakumar, [56] showed that administration of a methanolic gel extract of *A. vera* at doses of 1000, 2000, 4000, 8000, and 16000 mg/kg caused no mortalities or any changes in any of the investigated parameters at all the administered doses in the animals. Likewise, an aqueous leaf extract and supercritical carbon dioxide gel extract of *A. vera* caused no mortality or changes in the investigated parameters throughout the treatment period [57, 58, 76].

A study by Mwale and Masika [59] showed that an aqueous leaf extract of *A. ferox* at doses of 50, 100, 200, and 400 mg/kg caused a rise in the red blood cells, monocytes, and platelets counts and also hypertrophy of lungs, heart, and kidney and necrosis of spermatogenic cells in rats at all doses.

An ethanolic gel extract of *A. vera* at a dose of 100 mg/kg lowered the red blood cell count in addition to necrosis of the sex organs and hair loss around the genital area in male Swiss albino rats [65].

According to Koroye et al. [70], *Aloe vera* plus (GNLD) at doses of 0.2, 0.4, and 0.8 cm^3^ caused chronic inflammation, cell infiltration, necrosis, and fibrosis of the renal interstitium in all treated Wistar rats after 42 days of dosing.

Qmatrix® a product from *A. vera* leaves also caused an increase in absolute and relative kidney weight of males at 500 and 2000 mg/kg [77].

A 2-year study showed that an aqueous non-decolorized leaf extract of *A. vera* was found to increase the rates of hyperplasia of the stomach, small intestines, large intestines, and mesenteric lymph nodes in both rats and mice [78].

### Toxic compounds in the *Aloe vera and Aloe ferox*

Aloin, an anthraquinone present in both *A. vera* and *A. ferox*, has been associated with increased gastric motility causing diarrhea [79]. This explains why the *Aloe* species have been explored in relieving constipation. A study by Boudreau et al. [80] established that aloin caused pathological changes on the mucosa that were compared to those caused by *Aloe vera* whole leaf extract.

Aloe emodin, an anthraquinone present in *A. vera*, has been associated with hepatoxicity, genotoxicity, nephrotoxicity, phototoxicity, and reproductive toxicity [81–85].

### Potential for treatment of COVID 19

COVID 19 is caused by the Severe Acute Respiratory Syndrome coronavirus 2 (SARS-CoV-2). It belongs to RNA viruses and has four structural proteins (M (membrane), E (envelope), N (nucleocapsid), and S (spike)) [86]. The virus through its spike protein binds to the angiotensin-converting enzyme 2 (ACE2) receptors on the surface of the respiratory tract to facilitate its attachment and fusion with the host cell [86]. This is followed by entry into the host cell after priming of the S protein by the host cellular serine proteases TMPRSS2 [87]. The virus then releases its particles into the host cell, replicates, and invades the upper respiratory tract causing inflammation which later leads to acute respiratory distress. Treatment strategies involve use of antiviral drugs, immunomodulators, antibiotics, antioxidants, anti-inflammatory drugs, corticosteroids, and antipyretics [88–93]. Various medicinal plants including *Aloe vera* and *Aloe ferox* are being explored as potential drugs in the management of COVID 19 due to the various compounds they contain.

### Aloe vera

In silico studies have shown that anthraquinones including chrysophanol, aloe emodin, aloeresin, aloin A & B, 7-O-methylaloeresin, 9-dihydroxyl-2-O-(z)-cinnamoyl-7-methoxy-aloesin, and isoaloeresin are potential SARS-CoV-2 3CLpro protease inhibitors [94].

In addition, *Aloe vera* possesses anti-inflammatory activity [42, 60, 95–100] which helps in preventing the release of pro-inflammatory markers that cause inflammation which induces acute respiratory distress, the leading cause of mortality in COVID patients. *Aloe vera* also possesses immunomodulatory property [101–104], which strengthens the immune system of the host hence curbing the spread of the infection.

In addition, *A. vera* contains a phytosterol, β-sitosterol, with immunostimulatory activity helping to reinforce the host’s immune system. Molecular docking studies have shown that β-sitosterol strongly binds with the receptor-binding domain of the SARS-CoV-2 spike protein preventing the entry of the virus into the host cell [105].

Furthermore, *Aloe vera* contains mineral elements like zinc. Zinc has been found to inhibit the activity of corona RNA polymerase and SARS-coronavirus (SARS-CoV-2) replication in cell culture studies [106].

### Aloe ferox

In silico studies showed that anthraquinones (aloe emodin, aloinoside A, aloeresin D, Isoaloeresin A, etc.), phenolic compounds (pyrocatechol, p-Hydroxyacetophenone), and fatty acid derivatives (10-Hydroxyoctadecanoic acid, 10-Oxooctadecanoic acid) are potential SARS-CoV-2 main protease inhibitors [107].

Similar to *A. vera*, *A. ferox* is well endowed with anti-inflammatory compounds [108, 109]. These prevent the release of pro-inflammatory markers and cytokines that cause severe inflammation leading to acute respiratory distress in the patients.

## Conclusions

*A. vera* and *A. ferox* contain vast phytochemicals including anthraquinones, flavonoids, and phytosterols, which can be further studied for activity against SARS-CoV-2. Since herbal preparations made from *A. vera* and *A. ferox* are currently sold, this information will be used by the regulatory authorities before they issue marketing approval to the manufacturers of these products. More toxicity studies need to be carried out on the aqueous extracts of *A. vera* and *A. ferox* since decoctions are the most commonly used preparations by the local population. Also, more studies need to be done on the isolated compounds from these species so that they can be excluded from the preparations in case they are found to be toxic.

## Data Availability

Not applicable.

## Abbreviations

ESIMS: Electrospray ionization mass spectrometry
GC-MS: Gas chromatography-mass spectrometry
HPLC: High-performance liquid chromatography
HPLC-DAD: High-performance liquid chromatography with a diode-array detector
HPLC-MS: High-performance liquid chromatography-mass spectrometry
MALDI-TOF-MS: Matrix-assisted laser desorption/ionization-time of flight
MS: Mass spectrometry
NMRS: Nuclear magnetic resonance spectrometry
TLC: Thin-layer chromatography
TOF-MS: Triple quadrupole and time-of-flight mass spectrometry
